# Obscure gastrointestinal bleeding in a patient with neurofibromatosis type 1

**DOI:** 10.1111/ans.17573

**Published:** 2022-02-25

**Authors:** Ashok Gunawardene, Jesse Fischer

**Affiliations:** ^1^ Department of General Surgery Waikato District Health Board Hamilton New Zealand; ^2^ Department of Surgery University of Auckland Auckland New Zealand

A 54 year old man with neurofibromatosis type 1 (NF‐1) presented to the emergency department following four episodes of painless rectal bleeding with altered blood passed. Past history includes right adrenalectomy for phaeochromocytoma, a left optic nerve glioma and sciatica, for which he takes diclofenac regularly. The patient was not haemodynamically compromised with a blood pressure of 154/96 mmHg and initial haemoglobin was 162 g/L, falling to 74 g/L following fluid resuscitation. Urgent upper gastrointestinal endoscopy showed no source of bleeding and no further bleeding occurred in hospital. The patient had undergone colonoscopy within the last 2 years without abnormality, and in consideration of his diagnosis of NF‐1, outpatient CT enterography was arranged. This demonstrated hypervascular nodules in the small bowel, including a 3.5 cm lesion in the left upper quadrant and a 3.0 cm lesion in the central abdomen (Fig. 1). Following discussion in a multidisciplinary forum, small bowel resection was recommended.

**Fig. 1:**
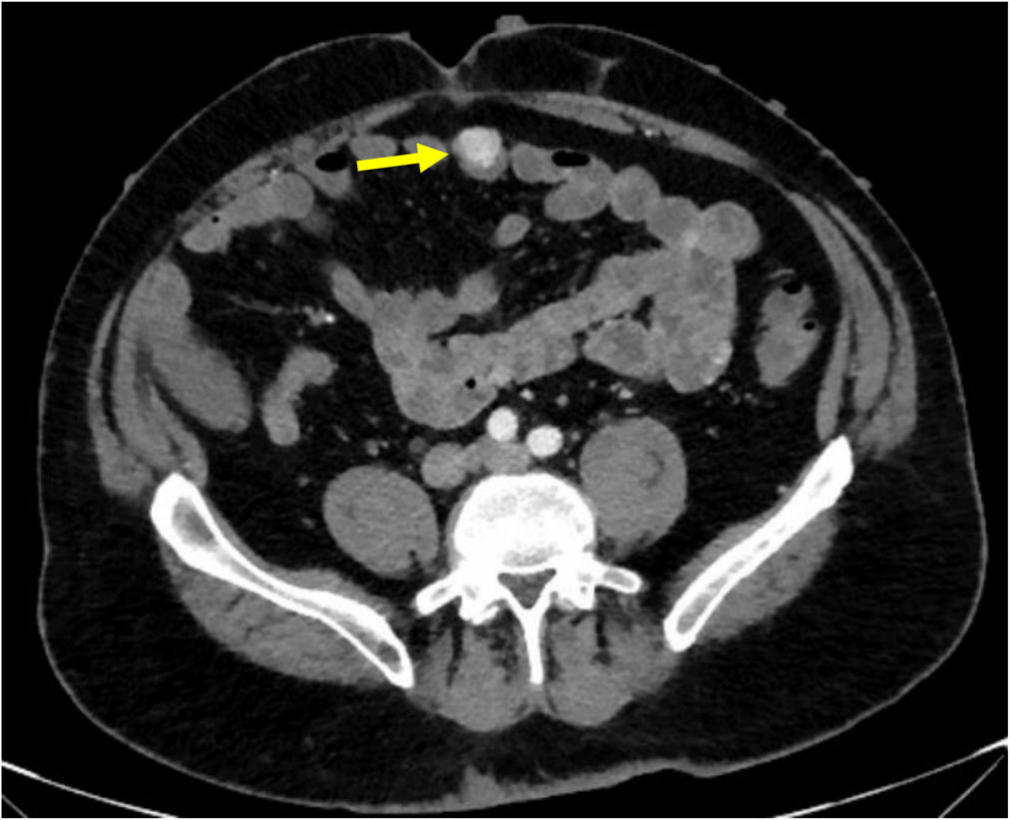
Axial slice CT abdomen in arterial phase demonstrating a hypervascular small bowel lesion in the mid‐abdomen (yellow arrow).

At laparotomy, over 20 nodules were identified in the small bowel. Two large mural nodules were noted in the mid‐jejunum (Fig. 2) consistent with the dominant lesions seen on imaging, and were resected along with a small number of adjacent lesions. With two primary anastomoses formed. The two largest exophytic lesions were also excised and the resulting seromuscular defects repaired with 3–0 PDS sutures. The remaining sub‐centimetre exophytic lesions were not excised. The patient was discharged on post‐operative day five after a short period of ileus requiring nasogastric decompression.

**Fig. 2:**
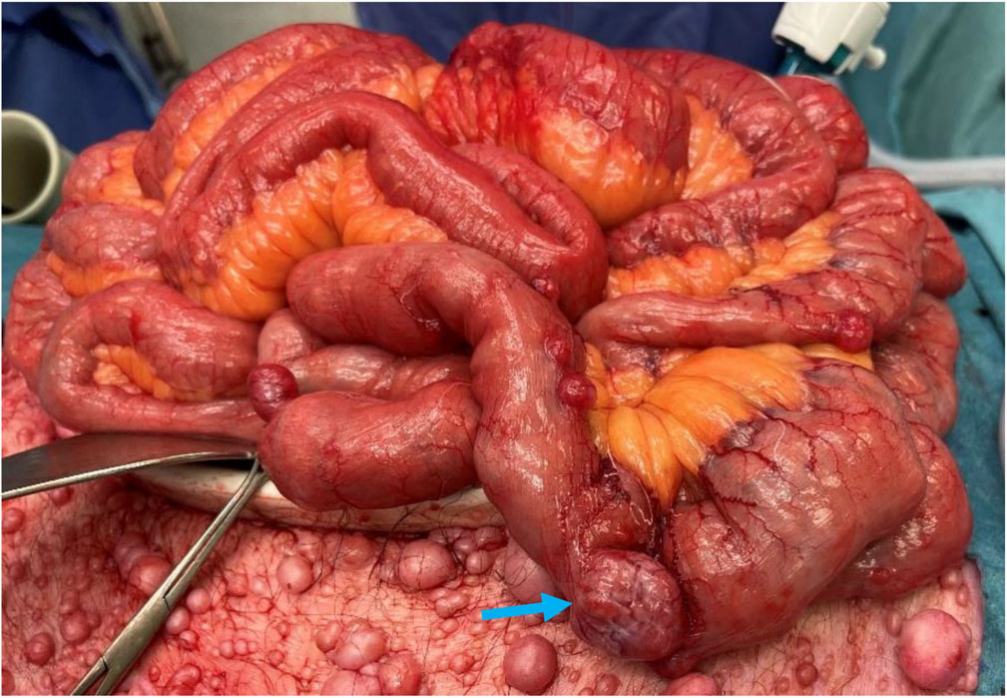
Intra‐operative photograph demonstrating numerous cutaneous neurofibromata, small exophytic small bowel nodules and a larger hyper‐vascular intra‐mural mass (blue arrow).

Pathological examination revealed multiple well‐circumscribed small bowel lesions formed by spindle cell proliferation with background collagen within the subserosal fat layer (Fig. 3). The spindle cells had bland nuclei, and stained positive for CD 117 and DOG1, consistent with gastrointestinal stromal tumours (GISTs). A very low replication index was noted on Ki 67 staining.

**Fig. 3:**
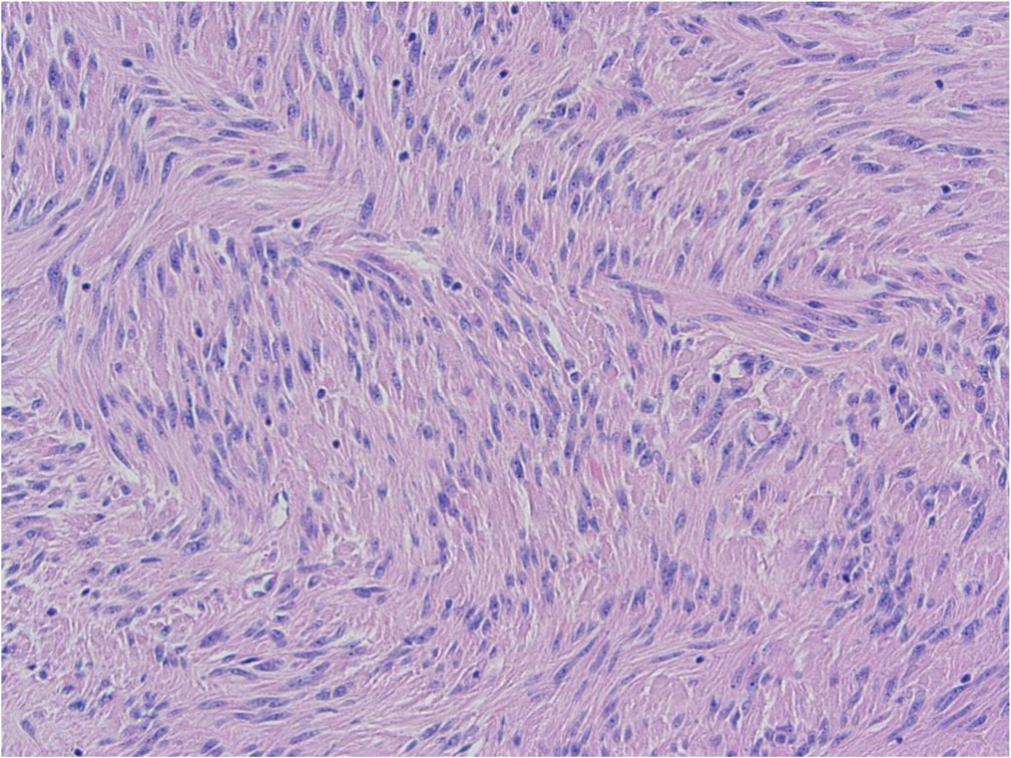
Haematoxylin and eosin section showing low‐grade spindle lesion.

Neurofibromatosis type 1 is an autosomal dominant inherited condition that results from a loss‐of‐function mutation in the NF‐1 gene that encodes Neurofibromin.^1^, ^2^ Gastrointestinal stromal tumours (GISTs), arising from Interstitial cells of Cajal,^3^ show an association with NF1, with an estimated lifetime risk of 7% among patients with NF‐1.^4^ While sporadic GISTs occur most frequently in the stomach (70% of cases), NF1‐associated GISTs (NF1‐GISTs) occur most frequently in the small bowel^5^ and exhibit a number of other distinct phenotypic features.^6^ These include a tendency to be multiple, spindle‐cell dominant and to have a low mitotic rate,^6^, ^7^ all features of the case reported. Risk factors for disease progression include a non‐gastric site, increasing size and mitotic rate greater than 5 mitoses per 50 high power fields.^8^

The primary indication for surgery in this case was gastrointestinal bleeding; secondary indications were to obtain histological diagnosis and prevent tumour progression. Although we could not be certain which lesion caused the patient's bleeding, we resected the two largest lesions, which were hypervascular and intra‐mural. We sampled the largest exophytic nodules for histological assessment. The risk of resecting all lesions with extensive small bowel resection, outweighed the benefit due to the indolent nature of small GISTs, especially in NF‐1 patients. This does however raise the question of how best to monitor both residual and new lesions. In this instance we elected to perform magnetic resonance enterography 12 months post‐operatively, with the expectation surveillance intervals could be increased if there was no radiological or clinical concern at that time. The optimal surveillance interval should be tailored to the individual based on histology, especially taking into account the site, size and mitotic rate. A number of risk stratification models exist to assist with this, including the National Institute of Health (NIH).^9^

Tyrosine kinase inhibitors such as imatinib have revolutionized the medical management of GISTs, but are dependent on c‐Kit/PDGFRA mutations to be effective.^10^ In a molecular analysis of NF1‐GISTs, c‐Kit/PDGFRA mutation was observed 8% of patients, in stark contrast to 80–90% of patients with sporadic GISTs.^10^ Mutational analysis should be performed, especially if imatinib may be indicated.^8^

In summary, small bowel GIST is an important differential diagnosis in obscure gastrointestinal bleeding in patients with NF‐1. NF‐1 GISTs possess unique characteristics compared with sporadic GISTs and are particularly indolent in nature. Treatment should be tailored to the individual but consideration to resection given when the diagnosis is in question, symptoms such as bleeding or bowel obstruction occur, or large lesions are seen on cross sectional imaging.

The patient gave consent to the writing‐up of this case report.

## Author Contributions

**Ashok Gunawardene:** Conceptualization; writing. **Jesse Fischer:** Conceptualization; writing.
